# Our Journal

**Published:** 1854-06

**Authors:** 


### OUR JOURNAL.

The next number will close the first year of the "Iowa Medical Journal." In truth we could not say that we, on the outset, regarded it as an experiment. We were assured that an honcst and faithful endeavor to make it useful and profitable would be appreciated by our medical brethren who have so generously given us "aid and comfort." Our subscription list has been daily increasing and we feel sufficient encouragement to push forward with the work. We hope to make the next volume still more interesting, for we contemplate some valnable improvements, and if our list were but a little larger, we would enlarge the work without any increase in the price of subscription. Let each subscriber consider himself a committee of one to get one more additional subscriber, and we would then materially enlarge. We have received numerous testimonials from our medical friends, of their appreciation of our effort to cater for their benefit, and from among these we select the following tribute, which is an extract of a letter from a medical friend in the State:

"It is now too late to say one word in reference to your excellent Journal. But I am constrained to say that under the circumstances I consider it one of the best papers for the Western practitioner that he can select. Short, practical, and to the point, the practical results of men of experience, just such a Journal as every western practitioner should subscribe for. Much of its matter is well calculated for the general reader, to guard him against the imposture and empiricism of QUACKS. Every portion of our country has, in many respects, its own medical topograhy, which cannot be so well understood (with the various modifications which it necessarily introduces into our practice) in any other way as from a faithful journal published in our midst, bringing fresh from the bedside of the patient such results as none other can supply. And while, from its size, it is not so full and minute as could be wished, yet I anticipate for it an increase of patrons, that will enable you to enlarge the work, and make it what I feel assured you can do, one among the very best Medical Journals of the 'West.' * * * *

"I am glad to learn that the School is in so fair a way to eminence and usefulness. With the talent, energy and enterprise of the Faculty, it cannot fail to rise very soon to the first rank among other similar institutions of our country. Any thing I can do to promote its success and increase its usefulness will be cheerfully done, and every good, sound medical man in the State should be animated by the same sentiments, and I trust they will act energetically in favor of its success."

